# Death of Dr. Moreton Stille

**Published:** 1855-09

**Authors:** 


### DEATH OF Dr. MORETON STILLE.

It is painful to record the death of a young physician so full of promise as was the subject of the following notice. The brother of this gifted young man is well known to the profession by his writings, and as Corresponding Secretary, for several years, of the American Medical Association. We take pleasure in transferring to our pages the obituary notice of Dr. Stillé in the Medical Examiner:

"Obituary.—Died, at Saratoga Springs, New York, on the 20th of August, Dr. Moreton Stillé, in the 33d year of his age.

"It is with sorrow that we announce the above melancholy event to our readers. Dr. Stillé's acquirements, manly character, unassuming deportment, and moral worth secured for him the respect and esteem of all who were acquainted with him. With most excellent natural abilities, he had a carefully cultivated mind, was master of several languages, and from advantages of study and travel, was both an accomplished scholar and physician. As a writer, besides occasional contributions to the American Journal of Medical Sciences, and to this Journal, Dr. Stillé was author of a most valuable paper on the pathology of Cyanosis, written for his thesis for the degree of Doctor of Medicine, and honored by being published by athority of the Faculty of the University of Pennsylvania. It is unnecessary for us to comment on the merits of that paper, as it is well known to the profession, and secured for its author, both at home and abroad, a reputation rarely attained by one of his early age.

"For the last two years of his life, Dr. S. had been unintermittingly occupied in writing a work on Medical Jurisprudence, which we are happy to state is printed, and will shortly be published. Early in the present year he was appointed Lecturer upon the Theory and Practice of Medicine in the Philadelphia Association for Medical Instruction. A rare critical sagacity applied to the results of extensive research rendered his instructions both accurate and complete, and we are informed by his colleagues that they were highly esteemed by the class to whom they were imparted.

There is every reason to think that the confinement and sedentary life, resulting from his deep interest in and close apglication to his posthumous work, together with the new and onerous duties duties of his Lectureship, were of great injury to a constitution and frame physically unfitted for such untiring labor.

"By his many friends his memory will long be warmly cherished, and we cannot cease to regret the bright promise blighted by his untimely death, we

"'Trust that those we call the dead
Are breathers of an ampler day
For ever nobler ends.'"

